# Targeting Irritable Bowel Syndrome Through Diet and Mechanism-Based Therapies: A Pathophysiological Approach

**DOI:** 10.3390/nu17223595

**Published:** 2025-11-17

**Authors:** Ioanna Aggeletopoulou, Katerina Karaivazoglou, Maria Kalafateli, Christos Triantos

**Affiliations:** 1 Division of Gastroenterology, Department of Internal Medicine, University of Patras, 26504 Patras, Greece; chtriantos@upatras.gr; 2 Department of Psychiatry, University of Patras, 26504 Patras, Greece; karaivaz@hotmail.com; 3 Division of Digestive Diseases, Department of Metabolism, Digestion and Reproduction, Faculty of Medicine, Imperial College London, London W2 1NY, UK; mariakalaf@hotmail.com

**Keywords:** irritable bowel syndrome, pathophysiology, FODMAP, personalized medicine, probiotics, psychobiotics, vitamin D

## Abstract

Irritable Bowel Syndrome (IBS) is a prevalent and heterogeneous functional gastrointestinal disorder with a complex and multifactorial pathophysiology. Traditional treatment approaches have focused on symptom relief, often overlooking the underlying biological mechanisms driving the disease. Τhis review summarizes the current evidence linking core pathophysiological pathways of IBS with mechanism- and diet- based therapeutic strategies to guide personalized treatment. Serotonergic signaling, microbial dysbiosis, immune activation, epithelial barrier dysfunction, and bile acid malabsorption interact to shape the diverse phenotypes of IBS, contributing to altered motility, visceral hypersensitivity, and gut-brain axis dysregulation. Increasing evidence supports that targeted dietary and biological interventions including low-FODMAP and Mediterranean low-FODMAP diets, targeted use of probiotics and psychobiotics, and vitamin D supplementation can modulate microbial composition, reduce luminal irritants, support barrier integrity, and attenuate immune system activation. Similarly, pharmacologic therapies including serotonergic receptor modulators, bile acid sequestrants and neuroimmune agents act on specific mechanistic pathways, reflecting a shift from symptom-based to mechanism-driven management. Collectively, these findings highlight that integrating dietary, microbial, neuroimmune, and serotonergic modulation within a unified therapeutic framework can support a more rational and individualized approach to IBS management and long term symptom control.

## 1. Introduction

Irritable Bowel Syndrome (IBS) constitutes a complex, multifactorial disease that affects millions of individuals worldwide, significantly impacting their quality of life [1]. IBS is a heterogeneous condition with a wide range of clinical presentations, characterized by chronic abdominal pain, bloating, and altered bowel habits [1,2]. The global prevalence of IBS in adults presents great variation across studies as it ranges between 5% and 15%, depending on the diagnostic criteria and the geographic region studied. Estimates based on stricter diagnostic criteria of Rome IV tend to fall between 3% and 5%, whereas broader, symptom-based studies report higher rates, typically between 10% and 15% [3,4,5]. According to the Rome IV criteria, IBS is classified into four subtypes: diarrhea-predominant IBS (IBS-D), constipation-predominant IBS (IBS-C), mixed-type IBS (IBS-M), and unclassified IBS (IBS-U) [1,6].

Despite its high prevalence, the management of IBS has been challenging due to its heterogeneous symptomatology and the incomplete understanding of its underlying pathophysiology. The disorder involves a complex interplay of mechanisms, including altered intestinal permeability, impaired modulation of gut motility, and increased visceral sensitivity [7,8]. In parallel, increasing evidence highlights the role of abnormal gut–brain axis (GBA) signaling and microbial dysbiosis, which can disrupt intestinal homeostasis and contribute to immune activation and low-grade inflammation [7,8]. Although various pharmacological and dietary interventions exist, most remain empirically guided and provide only partial or temporary relief in IBS symptoms. This highlights the need to bridge pathophysiological understanding with clinical management by targeting core biological mechanisms such as serotonergic signaling, immune activation, microbial imbalance, and barrier dysfunction, rather than symptoms alone.

Traditional treatment approaches have primarily focused on symptom relief that fails to address the underlying causes of the disease. However, growing evidence is unraveling the complex biological processes involved, paving the way toward the development of more targeted and personalized therapeutic strategies [9,10]. This review outlines current and emerging therapeutic strategies for IBS, focusing on how recent insights into disease mechanisms can guide more effective and individualized treatments. By highlighting the role of serotonergic signaling, gut microbiome, immune activation, and gut-brain interactions, the article emphasizes the importance of aligning clinical management with the evolving understanding of IBS pathophysiology. The central focus is to integrate mechanistic knowledge with therapeutic applications, providing a framework for personalized, mechanism-based IBS management.

## 2. Mechanistic Insights for Targeted IBS Therapies

### 2.1. Serotonergic Modulation and Receptor Targets

Serotonin is involved in regulating gut motility, secretion, and sensation, and its impaired regulation is associated with both IBS-D and IBS-C [11]. Approximately 95% of serotonin is synthesized by enterochromaffin cells within the gastrointestinal mucosa, and its bioavailability is strongly regulated by the serotonin reuptake transporter (SERT), which clears extracellular 5-HT from the synaptic space [12]. Dysregulation of this system via impaired enterochromaffin cell count or changes in tryptophan hydroxylase (TPH1/TPH2) expression, or polymorphisms affecting SERT gene have been associated with the pathophysiology of IBS [13]. Both IBS-D and IBS-C have been linked to reduced mucosal SERT expression, suggesting that impaired serotonin reuptake may disrupt motility regulation differently across different IBS phenotypes [14]. Subtype-specific receptor adaptations have also been observed, with upregulation of 5-HT3 receptors in IBS-D and downregulation of 5-HT4 receptors in IBS-C, suggesting that serotonergic modulation is both phenotype- and regionally-dependent in the gastrointestinal tract [15].

Serotonergic modulation remains a cornerstone of IBS treatment, with pharmacological agents targeting serotonin receptors offering significant symptom relief for many patients. To date, seven families of 5-HT receptors have been described, five of which (5-HT1, 5-HT2, 5-HT3, 5-HT4, and 5-HT7) are expressed in the intestinal tissue [16]. The 5-HT3 receptors are mainly localized on vagal afferents and intrinsic sensory neurons and mediate intestinal secretion, peristaltic reflexes, and visceral sensitivity [17]. Conversely, 5-HT4 receptors are expressed in neurons of the myenteric and submucosal plexuses, in primary afferent neurons, smooth muscle cells, and enterochromaffin cells of the gastrointestinal tract [18]. These distinct distributions explain the divergent roles of serotonergic agents across IBS subtypes, with 5-HT3 receptor upregulation predominating in IBS-D and reduced 5-HT4 activity contributing to constipation in IBS-C [15]. 5-HT3 receptor antagonists have demonstrated efficacy in reducing stool frequency, urgency, and abdominal pain in IBS-D, with recent randomized trials showing favorable safety profiles of ondansetron compared to earlier agents like alosetron [19]. Conversely, 5-HT4 receptor agonists, including prucalopride and mosapride, have shown beneficial effects in IBS-C by promoting intestinal transit and alleviating constipation symptoms [20,21]. Combination strategies coupling 5-HT4 agonists with probiotics showed higher clinical response rates, suggesting a potential microbiota-serotonin crosstalk influencing therapeutic efficacy [22]. Although earlier serotonergic agents such as alosetron were associated with rare cases of ischemic colitis [23] and cisapride or tegaserod with cardiovascular adverse events [24], subsequent development and clinical use of safer compounds have greatly reduced these risks [24,25]. Improved patient selection criteria and close monitoring have further minimized safety concerns, allowing targeted serotonergic therapies to be reintroduced with favorable risk-benefit profiles.

Beyond the use of receptor-targeting agents, selective serotonin reuptake inhibitors (SSRIs), which act peripherally to inhibit SERT, can increase synaptic 5-HT availability, and normalize intestinal motility [26]. SSRIs act by increasing serotonin concentrations in the enteric synapse, which enhances peristaltic reflexes and modulates sensory signaling [26]. Clinical studies indicate that SSRIs improve IBS symptoms and abdominal pain independently of their antidepressant effects [27]. They appear particularly useful in IBS-C, where they mildly enhance colonic contractility and tone, whereas tricyclic antidepressants (TCAs) are more effective in IBS-D due to their inhibitory effects on gut motility and visceral sensitivity [28]. Recent evidence also supports the modest but consistent efficacy of SSRIs in improving global IBS symptoms, while serotonin-norepinephrine reuptake inhibitors (SNRIs) have emerged as promising dual-action agents targeting both central pain perception and peripheral motility regulation [29,30,31]. These findings support the use of multimodal neuromodulators, extending beyond classical antidepressants.

Emerging evidence also highlights the 5-HT7 receptor as a novel therapeutic target. 5-HT7 receptor activation contributes to smooth muscle relaxation and modulation of bowel tone and sensitivity [32]. Research suggests that 5-HT7 antagonism in the acute phase may relax colonic smooth muscles, mitigating tension from gas and constipation in IBS-C, while also reducing bowel movement frequency to improve diarrhea in IBS-D [33]. Although preclinical findings indicate that 5-HT7 receptor modulation may alleviate visceral hypersensitivity and dysmotility, its clinical translation remains limited. No selective 5-HT7 receptor modulators have yet been approved for IBS, and evidence from human trials is lacking. Further studies are required to establish their safety, optimal dosing, and therapeutic efficacy before clinical implementation can be considered.

Collectively, serotonergic modulation includes a wide range of therapeutic mechanisms that align well with the evolving target of phenotype-specific and mechanism-based IBS treatment. The integration of genetic, microbial, and neuroendocrine factors in serotonergic research underscores the importance of a personalized therapeutic approach targeting the serotonin system in IBS management.

### 2.2. Gut Microbiome in IBS

The gut microbiome has emerged as a key player in IBS pathophysiology, with emerging evidence implicating microbial dysbiosis in the development and progression of the disorder [34]. Dysbiosis in IBS is typically characterized by a reduction in microbial diversity, an overgrowth of pathogenic bacteria, and a depletion of beneficial species [34]. Several factors contribute to the development and variability of gut dysbiosis in IBS, including dietary composition, antibiotic and proton pump inhibitor use, intestinal transit alterations, and host genetic background [35,36]. Distinct microbial patterns have been observed across IBS subtypes, with reduced *Bifidobacterium* and *Lactobacillus* levels more prominent in IBS-C and increased Enterobacteriaceae abundance in IBS-D [34]. These alterations likely reflect underlying differences in immune activation and mucosal permeability that shape microbial community structure [37,38]. This interindividual variability likely underlies the heterogeneous response to microbiota-targeted therapies, emphasizing the need for personalized modulation strategies. IBS patients commonly exhibit specific alterations in gut microbial composition, including an increased Firmicutes-to-Bacteroidetes ratio, reduced levels of beneficial genera such as *Bifidobacterium*, *Lactobacillus*, and *Faecalibacterium*, and an abundance of potentially harmful taxa, including members of the Enterobacteriaceae and Proteobacteria phyla (e.g., *Escherichia coli*) [34]. Contrary to the profound microbial depletion and inflammatory dysbiosis observed in inflammatory bowel disease (IBD) [39], the microbial imbalance in IBS is more subtle and functionally oriented, contributing to altered bile acid metabolism, short-chain fatty acid production, and low-grade immune activation rather than overt inflammation. Given that the gut microbiome can be modified through therapeutic and dietary interventions, translating these findings into clinical benefit will require larger and better-designed studies that consider diet and individual variability. Since diet strongly influences long-term microbiota stability, its careful evaluation could improve IBS management and also help clarify the biological links between IBS and IBD [40].

These microbial shifts can induce Toll-like receptor (TLR) signaling, notably TLR4 activation by Gram-negative bacterial lipopolysaccharides, triggering NF-κB activation in epithelial and immune cells [34]. This upregulates pro-inflammatory cytokines such as tumor necrosis factor-alpha (TNF-α), interleukin-6 (IL-6) and IL-1β, which disrupt tight junction proteins (occludin, claudins), impair barrier integrity, and perpetuate inflammation through microbial translocation [34]. These cascades contribute to immune activation, visceral hypersensitivity, and systemic inflammation observed in IBS.

Targeting these pathways, rifaximin, a non-absorbable antibiotic approved for IBS-D, reduces small intestinal bacterial overgrowth (SIBO) and dampens TLR-mediated inflammation, though repeated courses may be needed [41]. This has led to increased interest in microbiome-directed strategies aimed at restoring intestinal microbial balance. Probiotics, particularly those containing *Lactobacillus* and *Bifidobacterium* strains, have shown promise in alleviating IBS symptoms, likely through their ability to modulate immune responses, promote gut barrier function, and lessen visceral hypersensitivity [34]. While the evidence supporting the use of probiotics in IBS is still evolving, they represent a promising approach for personalized microbiome-based therapies.

Beyond probiotics, prebiotics and synbiotics have been explored as complementary strategies to modulate the gut microbiota in IBS. Prebiotics such as fructooligosaccharides and galactooligosaccharides selectively stimulate the growth of beneficial bacteria (e.g., *Bifidobacterium* species) [42] and improve IBS symptoms in experimental models [43]. Synbiotics combine these substrates with probiotic strains to enhance microbial stability and metabolic output [44]. In a recent randomized controlled trial (NCT05731232), a 12-week treatment with a multi-strain synbiotic significantly improved global and specific IBS symptoms compared with placebo, with 70% of patients achieving adequate relief and minimal adverse events [45]. Although current evidence suggests modest improvements in bloating and stool consistency, results remain heterogeneous [46], highlighting the importance of strain selection, dosage, and duration in optimizing efficacy.

Fecal microbiota transplantation (FMT) has also emerged as an innovative therapeutic strategy to restore microbial homeostasis in IBS [47]. Although some randomized trials have demonstrated improvements in stool consistency, bloating, and quality of life [48,49], others failed to show significant benefit over placebo [50]. Recent evidence supports the therapeutic potential of FMT in IBS. In a 2025 double-blind, randomized controlled trial, both encapsulated and rectal enema FMT significantly improved IBS symptom severity and quality of life compared with placebo, achieving clinical response rates of 86.7% and 73.3%, respectively [51]. These benefits were accompanied by increased microbial diversity and enrichment of beneficial taxa such as *Bifidobacterium longum* and *Dorea* spp., supporting the rationale for personalized microbiota-based therapy in IBS [51]. These inconsistencies likely reflect variability in donor selection, microbial composition, and delivery route, highlighting the need for standardized protocols and mechanistic studies before FMT incorporated into routine IBS management.

### 2.3. Immune Activation and Neuroimmune Pathways

Immune activation is increasingly recognized as a key driver of IBS pathophysiology, particularly in post-infectious IBS (PI-IBS), where a prior gastrointestinal infection triggers a persistent low-grade inflammatory response [52]. Mast cells play a central role in this process by releasing pro-inflammatory mediators such as histamine and tryptase that contribute to visceral hypersensitivity and altered gut motility [53]. Pro-inflammatory cytokines, such as IL-6 and TNF-α, further disrupt epithelial homeostasis, sensitize afferent neurons, and may impact the central nervous system via microglial priming, reinforcing visceral hypersensitivity [54].

Mast cell stabilizing drugs, such as ketotifen [55] and cromolyn sodium [56], have been investigated for their potential to alleviate IBS symptoms by inhibiting mast cell activation and the subsequent release of inflammatory mediators. Although some studies reported symptomatic improvement, particularly in pain and visceral hypersensitivity, their clinical benefit remains inconsistent, and these drugs are not currently established in routine IBS management. In addition, H1 receptor antagonists such as ebastine have demonstrated modest, but promising, effects in reducing abdominal pain and improving overall symptom relief in non-constipated IBS patients [57]. Although these agents are not widely used in clinical practice, they represent emerging immunomodulatory therapeutic approaches targeting the mast cell-nerve axis in IBS.

Beyond mast cell-targeted therapy, neuro-immune modulation through neurokinin receptor antagonists has also been explored. In a phase II randomized, double-blind trial of ibodutant, a selective neurokinin-2 receptor antagonist, in patients with IBS-D, female patients showed significant improvement over placebo in overall IBS symptoms and abdominal pain [58]. The treatment was well tolerated, with adverse event rates similar to placebo, supporting the potential of NK2 receptor blockade as a novel therapeutic strategy targeting neuro-immune interactions in IBS [58].

In addition, anti-inflammatory agents targeting the gut immune system have been explored as potential treatments for IBS. Mesalamine, a drug commonly used in IBD has been investigated in IBS patients with evidence of low-grade inflammation [59]. However, results have been inconsistent, and no clear consensus supports its routine clinical use. A recent meta-analysis indicates modest or negligible benefits, and current guidelines do not recommend mesalamine therapy as a standard of care [59].

Expanding beyond mesalamine, other immune-targeted therapies, such as mast cell stabilizers and NK2 receptor antagonists, have been evaluated in subgroups of IBS patients with mucosal immune activation or visceral hypersensitivity, particularly post-infectious and diarrhea-predominant types [58,59,60]. While preliminary results suggest symptomatic improvement, consistent biomarkers to guide patient selection remain lacking, as mast-cell counts and cytokine profiles show limited predictive value [61]. Collectively, these findings suggest that immune-targeted and anti-inflammatory therapies may play a role in selected IBS patients with documented immune activation, but further studies are needed to clarify their efficacy and safety.

### 2.4. Bile Acid Modulation and Gut Barrier Dysfunction

Bile acid malabsorption (BAM) is a common cause of diarrhea in IBS-D patients. Bile acids, which are normally reabsorbed in the terminal ileum, can accumulate in the colon, leading to watery diarrhea and abdominal cramping [62]. Beyond their detergent properties, bile acids act as signaling molecules via farnesoid X receptor (FXR) and G-protein coupled bile acid receptor (TGR5), influencing colonic transit, nociception, and mucosal immunity [63,64].

Recent randomized data demonstrate that the bile acid sequestrant colesevelam not only increases fecal bile acid excretion and hepatic bile acid synthesis but also modifies colonic mucosal gene expression, downregulating FXR and P2RY4, while upregulating TGR5 [65]. These findings suggest that bile acid sequestrant therapy (BAST) exerts molecular effects beyond simple bile acid binding, potentially influencing epithelial and immune signaling pathways. Such modulation may help explain the variable clinical response among IBS-D patients and underscores the interplay between bile acid metabolism and mucosal homeostasis. Supporting this evidence, experimental data from a chronic unpredictable mild stress (CUMS)- induced IBS model showed that cholestyramine, a bile acid sequestrant, reduced total and primary fecal bile acids, improved diarrhea and visceral hypersensitivity, restored gut barrier integrity, and ameliorated bone and muscle loss by enhancing osteoblast activity, downregulating muscle atrophy markers, and partially normalizing bile acid-transforming microbiota [66]. Interindividual variability in bile acids, ileal reabsorption efficiency, and gut microbial composition may further explain the heterogeneity in patient response to BAST [67,68]. While BAST is generally well-tolerated, it can cause side effects such as bloating and constipation, which may limit their use in some patients [62]. In parallel, there is growing interest in therapies that restore gut barrier integrity in IBS. Increased intestinal permeability has been observed in a subset of IBS patients and is thought to contribute to immune activation and systemic inflammation [69]. Gut barrier stabilizing therapies, such as butyrate and zonulin inhibitors, have shown promise due to their ability to induce epithelial barrier function and reduce intestinal permeability [70,71]. Butyrate, a short-chain fatty acid produced by the gut microbiota, not only has anti-inflammatory properties but also enhances histone acetylation, thereby upregulating tight junction proteins such as claudins and occludin, which are critical for maintaining barrier function [70,72]. Zonulin inhibitors, which are implicated in the regulation of tight junctions, are also being explored as potential therapies for microbiota-gut-brain axis disorders [71]. In this context, recent data highlight the potential of tenapanor, a minimally absorbed inhibitor of the sodium/hydrogen exchanger 3 (NHE3), which is approved for IBS-C. Beyond its effects on intestinal sodium handling and transit, tenapanor has been shown to strengthen intestinal barrier integrity and reduce visceral hypersensitivity [73]. In a preclinical experimental study, tenapanor attenuated cytokine- and IBS fecal supernatant-induced impairment in intestinal permeability, restored transepithelial electrical resistance in human colon monolayers, and reduced hyperexcitability of colonic sensory neurons through indirect modulation of TRPV1 signaling [74]. These findings support the hypothesis that enhancing epithelial barrier function may also mitigate abdominal pain by limiting paracellular antigen trafficking and downstream immune activation. In turn, restoring epithelial integrity with agents such as butyrate or tenapanor can indirectly modulate gut microbiota composition, as reduced luminal inflammation enhances recolonization by commensal species and strengthens mucosal homeostasis [75].

While these agents are still in the early stages of development, they represent promising approaches for targeting the underlying barrier dysfunction central to the pathophysiology of IBS.

### 2.5. Vitamin D as a Mechanism-Based Therapeutic Target in IBS

Vitamin D has emerged as a pleiotropic regulator in IBS, influencing immune modulation, epithelial integrity, and serotonergic signaling through genomic and non-genomic pathways [76]. The active form, 1,25-dihydroxyvitamin D3, binds to the vitamin D receptor (VDR), which is expressed throughout the gastrointestinal tract, modulating the transcription of genes involved in antimicrobial defense, tight junction assembly, and serotonin metabolism [77,78,79].

Vitamin D deficiency has been associated with increased intestinal permeability [80], gut microbiota dysbiosis [81], and enhanced Th1/Th17-driven inflammation [82], mechanistic insights which are also implicated in IBS pathophysiology. By suppressing pro-inflammatory cytokines (TNF-α, IL-6, IL-1β) and promoting the differentiation of regulatory T cells, vitamin D attenuates mucosal immune activation and may mitigate visceral hypersensitivity [76].

Beyond immune modulation, vitamin D directly affects serotonergic signaling by regulating tryptophan hydroxylase (TPH1/TPH2) and the SERT genes, thereby influencing motility and sensory responses along the GBA [83,84]. Calcitriol has also been shown to induce the expression of antimicrobial peptides such as cathelicidin and defensins, which enhance barrier function and restrain microbial overgrowth, mechanisms potentially relevant in IBS subtypes associated with dysbiosis [85].

Evidence from meta-analyses supports a modest but clinically relevant improvement in IBS Symptom Severity Score (IBS-SSS) [86,87] and quality of life [87,88,89,90] following vitamin D supplementation, particularly in patients with baseline deficiency. However, high heterogeneity and variable dosing regimens limit definitive conclusions. The observed benefits likely result from correcting vitamin D deficiency and thereby restoring epithelial integrity and neuroimmune homeostasis, rather than from supplementation beyond physiological levels [76].

Collectively, these data suggest that vitamin D acts as a mechanism-based adjunct therapy, restoring immune and neuroendocrine homeostasis in IBS. By shaping the gut microbiome and supporting intestinal health, vitamin D may serve as a therapeutic option, especially for deficient patients, representing a practical process toward personalized therapeutic management, though further research is needed.

## 3. Dietary and Nutritional Interventions

### 3.1. Low-FODMAP and Targeted Nutritional Strategies

Nutritional interventions have gained increasing interest as an option for managing IBS symptoms, particularly in patients with food intolerances or sensitivities. The low- fermentable oligosaccharides, disaccharides, monosaccharides, and polyols (FODMAP) diet has emerged as one of the most effective dietary strategies for IBS [91]. Fermentation of FODMAPs by colonic bacteria leads to rapid gas accumulation and short-chain fatty acid production, which can overstimulate chemosensing enteroendocrine cells intensifying luminal distension and discomfort [91,92].

Increasing evidence supports the hypothesis that reducing the intake of high-FODMAP foods leads to significant symptom improvement in many patients with IBS [91]. However, the low-FODMAP diet can be restrictive and difficult to adhere to, particularly without professional dietary guidance. Moreover, concerns have been raised regarding its potential impact on nutritional adequacy and the gut microbiome, as some studies suggest that prolonged adherence may reduce the abundance of beneficial bacterial species [91]. These observations are supported by data from meta-analyses, which have systematically assessed the efficacy of the low-FODMAP diet in IBS management. A meta-analysis assessed the effects of the low-FODMAP diet compared with standard IBS dietary advice on symptom severity and quality of life using the IBS-SSS [93]. The results demonstrated that adherence to a low-FODMAP diet leads to significantly greater improvement in both symptom control and quality of life than conventional dietary approaches [93]. Nonetheless, the authors highlighted potential drawbacks, including adverse alterations in gut microbiota composition and the risk of nutritional deficiencies, particularly when the diet is implemented without professional dietary supervision [93]. Consistent findings were reported by another meta-analysis which confirmed that the low-FODMAP diet resulted in significant reduction of abdominal pain and bloating [94]. However, the long-term superiority of this diet over traditional IBS dietary interventions remains uncertain. Similarly, Van Lanen et al., raised concerns regarding nutritional adequacy and the possible negative impact on the gut microbiome with prolonged adherence [95]. Consistent with these findings, a network meta-analysis demonstrated that the low-FODMAP diet outperforms other dietary interventions in improving overall IBS symptoms. However, most of the included trials were conducted in secondary or tertiary care and did not evaluate the effects of FODMAP reintroduction or dietary personalization on symptom outcomes [96].

While the low-FODMAP diet has been shown to be highly effective in reducing IBS symptoms, its successful implementation in clinical practice requires individualized guidance, particularly during the reintroduction and personalization phases to ensure long-term sustainability and dietary balance. Increasing attention is now being directed toward personalized nutritional approaches that consider individual symptom patterns, gut microbiota composition, and psychosocial factors, aiming to optimize therapeutic outcomes and enhance quality of life in IBS patients.

### 3.2. Psychobiotics and Microbiota-Gut-Brain Axis Modulation

The GBA, a bidirectional communication between the gut and the central nervous system, plays a critical role in IBS pathophysiology. Dysregulation of this system is thought to contribute to visceral hypersensitivity, altered gut motility, and psychological comorbidities commonly observed in IBS patients [97]. Psychobiotics are a distinct subclass of probiotics that act primarily through the GBA [98,99]. Unlike conventional probiotics, which mainly enhance gastrointestinal health, psychobiotics play a key role in neurotransmission, immune balance, and neuroendocrine signaling, thereby modulating behavior and stress responses [100]. Psychobiotics, have emerged as a novel therapeutic approach for targeting GBA dysfunction in IBS [101]. These probiotics, which include genera such as *Lactobacillus* and *Bifidobacterium*, have been shown to modulate the gut microbiota, reduce systemic inflammation, and influence neurotransmitter production, including serotonin and gamma-aminobutyric acid [101]. Psychobiotics may also influence central nervous function via modulation of the hypothalamic-pituitary-adrenal (HPA) axis and vagal tone, while promoting intestinal barrier integrity and short-chain fatty acid production [102]. Βy shaping tryptophan metabolism, they can shift the balance toward serotonin rather than neurotoxic kynurenines, potentially alleviating both intestinal and mood symptoms [103].

Clinical studies have demonstrated that psychobiotics can improve both gastrointestinal and psychological symptoms in IBS patients, including improvements in anxiety, depression, and stress [101,104]. *Lactiplantibacillus plantarum* 299v, *Bifidobacterium longum* R0175, and *Lactobacillus helveticus* R0052 have shown particular efficacy in reducing abdominal pain, bloating, and stress-related gastrointestinal symptoms, accompanied by improvements in mood and quality of life [101]. A recent meta-analysis confirmed that probiotic supplementation improves quality of life in IBS, although psychobiotic-specific effects on anxiety and depression require further investigation [105]. While the mechanisms underlying these effects are not completely understood, psychobiotics represent a promising mechanism-based adjunct therapy targeting the complex interplay between the gut and the brain in IBS. However, further research is needed to identify the most effective strains, dosages, and treatment durations for psychobiotic therapy in IBS.

### 3.3. Combined and Personalized Dietary Therapies

Given the multifaceted mechanisms underlying IBS, monotherapies often provide only partial symptom relief. As a result, increasing interest has been directed toward combined or integrative therapeutic approaches that combine various pathophysiological mechanisms. The combination of dietary interventions, pharmacological treatments, and GBA modulating strategies, such as probiotics and psychological therapies, has shown promising results in enhancing symptom control, improving quality of life, and promoting long-term disease management. Evidence from a clinical trial that implemented a four-week dietary intervention comparing a low-FODMAP and traditional dietary advice (LFTD) diet, a low-carbohydrate diet, and optimized medical treatment in patients with IBS, showed that both active diet groups achieved substantial reductions in symptom severity, with the greatest improvement observed in the combined dietary arms [106]. This evidence enhances the idea that integrating low-FODMAP approaches with conventional dietary guidance can enhance clinical outcomes. Recent evidence supports a modified approach combining the Mediterranean and low-FODMAP diets [107]. A randomized controlled trial showed that the Mediterranean low-FODMAP diet (MED-LFD) led to greater improvements in IBS symptom severity and quality of life compared to a diet based on the National Institute for Health and Care Excellence (NICE) guidelines [107]. Beyond symptomatic relief, the combined approach was associated with favorable modulation of gut microbial metabolites, including short-chain and branched-chain fatty acids, suggesting a beneficial impact on gut microbial activity and intestinal homeostasis. Importantly, adherence rates were higher and no adverse effects were reported, indicating that incorporating Mediterranean dietary principles may enhance both the efficacy and sustainability of low-FODMAP interventions [107]. In a pilot randomized controlled trial comparing the Mediterranean diet versus a low-FODMAP diet in patients with non-constipated IBS (IBS-D or IBS-M), 81.8% of participants in the low-FODMAP group and 73% in the MD group achieved ≥30% reduction in abdominal pain [108]. The LFD group also showed a greater reduction in IBS-SSS scores [108].

Beyond therapeutic management, recent data suggest that adherence to healthy dietary patterns may also influence IBS risk. In a recent matched case-control study, higher adherence to the Mediterranean diet and greater diet quality, as reflected by the Prime Diet Quality Score (PDQS), were both independently associated with significantly lower odds of newly diagnosed IBS [109]. These findings support the hypothesis that Mediterranean-type dietary patterns may exert a protective role against IBS development, possibly through modulation of gut microbiota, inflammation, and neuroendocrine function.

In addition, regular physical activity has been shown to exert beneficial effects in IBS through its impact on both gastrointestinal and psychological aspects. Lindsell et al. reported that routine exercise was associated with a 15–66% reduction in IBS symptom severity and up to a 41% improvement in quality of life, supporting the hypothesis that these benefits may be mediated by favorable modulation of the gut microbiome [110]. Prospero et al. further demonstrated that a 12-week aerobic exercise program significantly improved gastrointestinal and psychological symptoms in IBS patients, regardless of psychological profile, underscoring the value of incorporating physical activity into IBS management [111]. Collectively, these findings support physical activity as a safe, non-pharmacological adjunct that may enhance both gut and mental health in IBS.

Lastly, emerging evidence supports the adjunctive use of several natural and plant-based therapies in IBS [112]. Peppermint oil, rich in menthol, exerts smooth muscle-relaxing and antispasmodic effects via calcium channel blockade and has shown consistent benefits in reducing abdominal pain and bloating [113]. Herbal combinations such as Iberogast (STW 5) demonstrate symptom improvement through modulation of motility and visceral sensitivity [114]. Curcumin has also been investigated for their anti-inflammatory and antioxidant properties, with modest improvement in symptom severity and good safety profile [115]. Overall, phytotherapeutic agents appear to provide mild-to-moderate symptom relief, particularly for pain and bloating, but their efficacy depends on formulation, dosage, and IBS subtype. Further large-scale randomized studies are needed to confirm their long-term safety and mechanistic specificity.

### 3.4. Toward Mechanism-Based and Personalized Treatment

The integration of dietary, pharmacologic, and microbiome-based therapies reflects a core shift from symptom management toward mechanism-based treatment. Dietary strategies such as the low-FODMAP or Mediterranean low-FODMAP diets, vitamin D supplementation, and targeted prebiotic or psychobiotic use may act synergistically to modulate microbiota composition, restore barrier integrity, and reduce immune activation. Table 1 presents a concise overview of the therapeutic modalities for IBS, highlighting their principal mechanisms and clinical effects.

**Figure 1:**
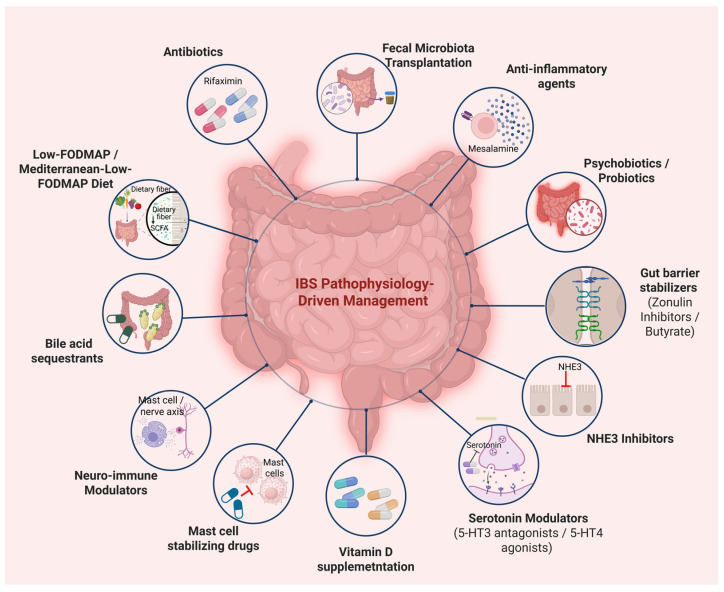
Pathophysiology-Driven Management Strategies for Irritable Bowel Syndrome.

These approaches can be used in combination with pharmacological agents that specifically target serotonergic, bile acid, or neuroimmune pathways. Considering the biological heterogeneity of IBS, therapy should be individualized according to the dominant mechanistic pathway, including dysbiosis, barrier dysfunction, altered serotonin signaling, or immune activation, with guidance from emerging biomarkers and clinical phenotyping. As depicted in Figure 1, this pathophysiology-driven model connects distinct biological mechanisms with corresponding therapeutic interventions, establishing an integrated framework for personalized IBS management.

Serotonin modulators (5-HT3 antagonists/5-HT4 agonists) regulate impaired motility and visceral sensitivity. Rifaximin and probiotics/psychobiotics target microbial dysbiosis, modulate gut-brain signaling, and restore microbial homeostasis. Fecal microbiota transplantation (FMT) represents an emerging microbiota-directed therapy. Anti-inflammatory agents and mast cell-targeted drugs reduce mucosal inflammation and neuroimmune activation and neurokinin receptor antagonists further attenuate hypersensitivity. Bile acid sequestrants, NHE3 inhibitors, butyrate, and zonulin inhibitors enhance barrier integrity and fluid balance. Vitamin D supplementation supports barrier integrity and immune modulation. Dietary interventions such as the low-FODMAP or Mediterranean–low-FODMAP diet reduce luminal fermentation and distension, alleviating symptoms in sensitive individuals. These interventions reflect a shift toward mechanism-based and individualized management of IBS. Created with BioRender.com (accessed on 20 October 2025). Abbreviations: IBS, irritable bowel syndrome; FODMAP, fermentable oligosaccharides, disaccharides, monosaccharides and polyols; SCFA, short chain fatty acid; 5-HT, 5-hydroxytryptamine (serotonin); NHE3, sodium/hydrogen exchanger 3.

## 4. Future Perspectives

Understanding the underlying mechanisms of IBS highlights the importance of moving toward a more targeted and individualized treatment approach. Future management is expected to rely less on empirical strategies and more on identifying the predominant biological pathways involved, including microbial, neuroendocrine, immune-mediated, or barrier-related, enabling more precise therapeutic targeting. Incorporating mechanistic biomarkers in clinical practice could support better treatment selection and help guide the design of more focused clinical trials. At the same time, advances in multi-omics technologies such as microbiome profiling and molecular diagnostics may allow for more accurate monitoring of treatment response. Gradually incorporating these tools into routine care will help establish a personalized framework for IBS management, offering the potential for more durable symptom control and improved quality of life for affected individuals.

## 5. Conclusions

Recent advances in IBS research reveal a complex interplay of pathophysiological mechanisms driving symptom heterogeneity. Dysregulation of serotonergic signaling, particularly through 5-HT receptor subtypes and altered SERT expression, contributes to abnormal motility and visceral hypersensitivity across IBS subtypes. Microbial dysbiosis, characterized by reduced diversity and overgrowth of pathobionts, activates TLR pathways, impairing barrier integrity and promoting low-grade inflammation, while bile acid malabsorption exacerbates diarrhea and disrupts FXR and TGR5 signaling. Immune activation, marked by mast cell mediators and pro-inflammatory cytokines, further sensitizes the gut-brain axis. Disrupted epithelial tight junctions allow antigen translocation, sustained by zonulin and mitigated by microbial metabolites like butyrate. Nutritional interventions such as the low-FODMAP diet reduce luminal triggers but may alter microbiome composition. Psychobiotics offer emerging tools to restore gut-brain communication and serotonin balance via modulation of tryptophan metabolism.

While challenges remain in identifying reliable biomarkers and optimizing treatment selection, the future of IBS therapy holds great promise for improving patient outcomes and achieving long-term outcomes. Aligning treatment strategies to distinct subtypes of IBS represents a significant advancement toward personalized therapy, potentially transforming the management of this complex disorder.

This review has certain limitations. Most of the studies discussed are heterogeneous in terms of design, and patient populations, which limits direct comparison of results. Moreover, the present review is narrative rather than systematic, and the interpretation of emerging mechanisms and therapeutic implications should be considered within this context.

In conclusion, bridging mechanistic understanding with clinical application through mechanism-based and personalized treatment models may ultimately transform IBS from a symptom-defined disorder into a biologically stratified, precision-managed condition.

## Figures and Tables

**Table 1:** Summary of Mechanism-Based and Dietary Therapeutic Approaches in IBS.

Therapeutic Category	Representative Interventions	Mechanistic Target/Pathway	Key Findings
Serotonergic Modulation	5-HT3 antagonists (ondansetron, alosetron); 5-HT4 agonists (prucalopride, mosapride); SSRIs, SNRIs	Regulation of gut motility, visceral sensitivity, serotonin reuptake	Improves stool frequency, urgency, abdominal pain in IBS-D; enhances colonic transit in IBS-C; neuromodulators show consistent but modest benefit
Gut Microbiome–Directed Therapies	Rifaximin, probiotics, prebiotics, synbiotics, FMT	Modulation of dysbiosis, TLR signaling, and microbial homeostasis	Rifaximin reduces bacterial overgrowth and inflammation; probiotics and synbiotics improve IBS symptom scores; FMT increases microbial diversity with variable efficacy
Immune and Neuroimmune Modulators	Ketotifen, cromolyn sodium, ebastine, ibodutant, mesalamine	Mast cell stabilization, cytokine inhibition, NK2 receptor blockade	Reduce visceral hypersensitivity and abdominal pain in selected patients; clinical benefit remains inconsistent across studies
Bile Acid and Barrier–Targeted Therapies	Colesevelam, cholestyramine, butyrate, zonulin inhibitors, tenapanor	FXR/TGR5 signaling, epithelial integrity, intestinal permeability	Bile acid sequestrants improve stool consistency and barrier function; butyrate and tenapanor strengthen epithelial junctions and reduce hypersensitivity
Vitamin D Supplementation	Vitamin D_3_ (cholecalciferol)	Serotonin metabolism, barrier and immune regulation	Improves symptom severity and quality of life, particularly in vitamin D deficient individuals
Dietary Interventions	Low-FODMAP, Mediterranean–Low-FODMAP diets	Reduction of luminal fermentation and osmotic load	Reduce bloating and abdominal pain; associated with favorable modulation of gut microbial metabolites
Lifestyle Measures	Regular physical activity	Gut–brain axis modulation	Decreases IBS symptom severity and improves quality of life
Natural Products and Phytotherapy	Peppermint oil, Iberogast (STW 5), Curcumin	Smooth muscle relaxation; modulation of motility and visceral sensitivity; anti-inflammatory effects	Provide mild-to-moderate symptom relief; improve motility and comfort; good safety profile; variable efficacy

Abbreviations: 5-HT, 5-hydroxytryptamine; SSRI, Selective Serotonin Reuptake Inhibitor; SNRI, Serotonin-Norepinephrine Reuptake Inhibitor; IBS, Irritable Bowel Syndrome; FMT, Fecal Microbiota Transplantation; TLR, Toll-like Receptor; NK2, Neurokinin-2 Receptor; FXR, Farnesoid X Receptor; TGR5, Takeda G-protein-coupled Receptor 5; FODMAP, Fermentable Oligo-, Di-, Mono-saccharides And Polyols.

## Data Availability

This article is a review and does not report any new data.
